# Endoplasmic Reticulum Stress-Mediated Basolateral Amygdala GABAergic Neuron Injury Is Associated With Stress-Induced Mental Disorders in Rats

**DOI:** 10.3389/fncel.2019.00511

**Published:** 2019-11-14

**Authors:** Songjun Wang, Weibo Shi, Guozhong Zhang, Xiaojing Zhang, Chunling Ma, Kai Zhao, Bin Cong, Yingmin Li

**Affiliations:** Hebei Key Laboratory of Forensic Medicine, Department of Forensic Medicine, Collaborative Innovation Center of Forensic Medical Molecular Identification, Hebei Medical University, Shijiazhuang, China

**Keywords:** stress, amygdala, GABAergic neurons, mental disorder, endoplasmic reticulum stress

## Abstract

The amygdala is an important center of fear learning and memory and plays a critical role in regulating stress disorders. Previous studies have shown that changes in the amygdala caused by stress are an important cause of mental disorders including anxiety, but the specific mechanism remains unclear. Therefore, the purpose of this study was to investigate whether mental disorders induced by stress are related to γ-aminobutyric acid (GABA)ergic neuron damage in the basolateral amygdala (BLA) and whether endoplasmic reticulum stress (ERS) is involved in the injury process. Rat models of different durations of stress were established by restraint and forced ice-water swimming. Behavioral tests and high-performance liquid chromatography (HPLC) were used to detect anxiety in rats and changes in neurotransmitter levels in the BLA. Morphological approaches and microscopy-based multicolor tissue cytometry (MMTC) were used to detect the damage-induced changes in GABAergic neurons in the BLA. Immunofluorescence double labeling was used to detect the expression of ERS-related proteins before and after the inhibition of protein kinase R-like endoplasmic reticulum kinase (PERK) pathway. Stress resulted in damage to GABAergic neurons in the BLA, decreased GABA and increased glutamic acid (GLU) levels, perturbation of the excitation/inhibition (E/I) ratio in the BLA, and obvious anxiety disorders in rats. Moreover, ERS-mediated GABAergic neuron injury was an important cause of neurotransmitter level changes in the BLA. These results suggested that ERS-mediated GABAergic neuron injury in the BLA may be an important cause of stress-induced mental disorders.

## Introduction

Stress involves a series of neuroendocrine, physiological and pathological reactions that occur when the body is stimulated by internal and external factors (McEwen, 2007). Moderate stress enhances the body’s ability to resist external risk factors, while excessive stress damages the body, affects normal psychological and physiological functions (Tsigos and Chrousos, 2002), and results in pathological changes in important organs of the body (Puga et al., 2015; Ye et al., 2017; Yi et al., 2017; Zheng et al., 2018; Kaczmarek et al., 2019). As broadly documented in clinical and animal literature (Caruso et al., 2018), stress exposure is a crucial risk factor for the development of mental disorders, including anxiety disorders and major depression. Additionally, the length of stress exposure is related to the degree of impairment of mental disorders.

The amygdala is a key brain region for the acquisition and processing of learned fear associations, and plays a critical role in regulating stress-induced disorders, including anxiety disorders (LeDoux, 2000; Davis and Whalen, 2001). The basolateral amygdala (BLA), consisting of approximately 80% glutamatergic neurons and 20% γ-aminobutyric acid (GABA)ergic neurons (Davis et al., 1994; Chauveau et al., 2012), is an important subnucleus of the amygdala. The BLA projects to the central nucleus of the amygdala (CeA), which mediates autonomic and behavioral responses associated with fear and anxiety *via* further projections to the brainstem (Davis, 2000). Although the primary output region of the amygdala is the CeA, activation of the CeA is dependent on the glutamatergic projections of the BLA. Previous studies on the BLA mainly focused on glutamatergic neurons and seldom investigated GABAergic neurons in the BLA, mostly because the BLA is primarily composed of glutamatergic neurons. Patients with stress-induced anxiety disorder have abnormally increased activity levels in the BLA (Etkin et al., 2009). However, it is still unclear whether GABAergic neurons in the BLA are involved in this process and affect the glutamatergic projections from the BLA to the CeA.

Perturbations causing instability of the endoplasmic reticulum (ER) result in the accumulation of unfolded and misfolded proteins in a pathological process known as endoplasmic reticulum stress (ERS; Boyce and Yuan, 2006). When ERS occurs, cells respond to unfolded and misfolded proteins by initiating a series of complex signal transduction cascades, namely the unfolded protein response (UPR). The UPR is a cellular process that is highly conserved across species and that functions to restore and enhance the ability of the ER to process proteins and to avoid the disastrous outcome of uncontrolled and excessive accumulation of unfolded and misfolded proteins. Protein kinase R-like endoplasmic reticulum kinase (PERK) is an important sensing element for ERS and a trigger of the PERK-eukaryotic translation initiation factor 2α (eIF2α)-activating transcription factor (ATF4)-CHOP pathway (Hetz, 2012). When ERS is prolonged and/or severe, the ERS PERK-eIF2α-ATF4-CHOP pathway will be activated and induce cell death (Kang et al., 2017; Yi et al., 2019).

Given the important relationship between stress disorders and the BLA, we established rat models of different durations of stress exposure and focused on GABAergic neuron injury in the BLA and dynamic changes in ERS-related proteins. The purpose of this study was to determine: (1) whether stress disorders are associated with damage to GABAergic neurons of the BLA; and (2) whether ERS is involved in this damage.

## Materials and Methods

### Animals

Male Sprague–Dawley (SD) rats (Experimental Animal Center, Hebei Medical University, China), weighing 220 ± 20 g, were bred (4/cage) and housed on a 12/12-h light/dark cycle in a temperature- and humidity-controlled room. The rats were given *ad libitum* access to food and water. Rats were randomly assigned to the following groups: control group; restraint stress combined with ice water swimming (stress) groups at 1, 3, 7, 14 and 21 days. Additionally, to investigate the effects of ERS on GABAergic neurons of the BLA under stress exposure, we also included a group exposed to stress that was treated with the PERK pathway inhibitor salubrinal for 7 days (stress+sal) and a group that was only treated with salubrinal for 7 days (sal; *n* = 10 rats per group). All procedures followed the National Institutes of Health guidelines and were approved by the Institutional Review Board for Animal Experiments at Hebei Medical University.

### Animal Treatments and Experimental Procedure

The restraint stress and ice-water swimming protocols were performed as previously described (Yi et al., 2017). Briefly, rats were placed in a restraint device with no food and water for 6 h (from 8:00 AM to 14:00 PM) each day. Then, the restrained rats were placed in ice water to swim for 5 min each day. The stress process lasted for 1, 3, 7, 14, or 21 days. The control group rats were left in the cages for the same amount of time with no food or water. For the stress+sal group, rats were injected intraperitoneally (i.p.) with the PERK pathway inhibitor salubrinal (1 mg/kg i.p.) half an hour before stress treatment. For the sal group, rats were only injected with salubrinal (1 mg/kg i.p.). The protocol for the stress+sal and sal groups was performed for 7 days.

### Behavioral Experiments

#### Open Field Test

An open field test was performed under dim red lights (28 lux) using an acrylic box (40 cm × 40 cm × 65 cm), and the activity of rats was monitored by an automated activity monitoring system (Tru Scan^TM^, Photobeam Sensor-E63-22, Coulbourn Instruments, Whitehall, PA, USA). The test lasted 10 min. The percentage of movement distance in the center area [(Movement distance in the center area/Movement distance in the overall area) × 100] and the percentage of cumulative duration in the center area [(Cumulative duration in the center area/Cumulative duration in the overall area) × 100] were used as measures of anxiety-like behavior.

#### Elevated Plus-Maze (EPM)

The elevated plus-maze (EPM) consisted of two open arms (50 cm × 10 cm), two closed arms (50 cm × 10 cm × 40 cm) and a central area (10 cm × 10 cm) The Plexiglas arms were elevated 50 cm above the ground. Behavioral testing was performed under dim red lights (28 lux). Each rat was recorded by an overhead camera, and behavior was scored for 5 min by an automated video tracking system (ANY-maze v.4.6, Stoelting, Wood Dale, IL, USA). The percentage of time spent in the open arms [Time in open arms/(Time in open arms + Time in closed arms)] × 100 and the percentage of open arm entries [Open arm entries/(Open arm entries + Closed arm entries)] × 100 were used as measures of anxiety-like behavior, and the number of closed arm entries was used as a measure of general locomotor activity (de Oliveira Citó Mdo et al., 2012).

### Tissue Preparation

Sixty minutes after the behavioral tests, the rats were deeply anesthetized and sacrificed. Brains used for staining were harvested and fixed immediately in 10% formalin. After subsequent dehydration in a graded ethanol series and embedding in paraffin, brain sections (5 μm) beginning at −1.80 mm from the Bregma, based on a stereotaxic atlas, were obtained (Paxinos and Watson, 2007). Then, the sections were prepared for thionin staining, immunohistochemistry, and immunofluorescence double staining. In other rats, brains were rapidly removed and stored immediately at −80°C until measurements of glutamic acid (GLU) and GABA in the BLA were performed.

### Estimation of GLU and GABA Levels in the BLA

Tissue concentrations of GLU and GABA were measured by high-performance liquid chromatography (HPLC) with a fluorescence detector (Chen et al., 2019). Samples of the BLA (10 mg) were homogenized in saline solution (1 g: 9 ml). Twenty microliter of BLA homogenate was mixed with 70 μl 0.4 M perchloric acid, and then centrifuged at 12,000 *g* and 4°C for 20 min. The supernatants were removed for analysis and filtered through a 0.22 mm GV filter (Millipore, Bedford, MA, USA). Then, 10 μl of each sample was injected into an Agilent Poroshell HPH-C18 column (4.6 mm × 50 mm, 2.7 μm) through a temperature-controlled autosampler (Agilent). The mobile phase was composed of 10 mM disodium hydrogen phosphate buffer and 10 mM sodium borate buffer in water, methanol and acetonitrile (78:13:9, v/v). The flow rate was kept constant at 1 ml·min^−1^. Chromatographic analyses were performed at the column temperature of 35°C. Fluorescence detector as operated using an excitation wavelength of 355 nm and an emission wavelength of 450 nm. Methanol was used as an internal standard, and the neurochemicals of interest were quantified using HP ChemStation software (Agilent, St. Clara, CA, USA). The excitation/inhibition (E/I) ratio was defined as the GLU/GABA ratio.

### Immunohistochemistry

According to the protocol recommended by the immunohistochemistry kit, deparaffinized sections were pretreated using microwave antigen retrieval, followed by incubation in 3% H_2_O_2_ in methanol for 30 min and then incubation in goat serum for 30 min. Next, tissues were incubated with a polyclonal antibody specific for mouse GAD65/67 (1:100) overnight at 4°C. The tissues were then incubated for 1 h with a biotinylated secondary antibody and subsequently incubated with horseradish peroxidase (HRP)-conjugated biotin for 30 min. Finally, 3,3′-diaminobenzidine (DAB) was used as the chromogen. Tissues were counterstained with hematoxylin.

### Immunofluorescence Double Staining

Immunofluorescence double staining was performed as described previously (Shi et al., 2019). Monoclonal antibodies against p-eIF2α (1:100), ATF4 (1:100) or CHOP (1:100) were used as the first primary antibodies, and polyclonal anti-GAD65/67 antibody (1:100) was used as the second primary antibody. DyLight^TM^ 488-Conjugated AffiniPure Goat Anti-Mouse IG (1:100) and DyLight^TM^ 594-Conjugated AffiniPure Goat Anti-Rabbit IG (1:150) were used as secondary antibodies.

### Cell Counting

For GAD65/67^+^ cells, cell counting was performed as described previously (Yi et al., 2017). Five rats from each group were used for morphological observation and data analysis. The largest amygdala area was accurately identified according to the stereotaxic atlas (Paxinos and Watson, 2007). Using the serial section technique, one of every five sections was selected for a total of three sections from each rat. Microscopy-based multicolor tissue cytometry (MMTC) has been widely used to quantify DAB-positive cells (Hamzei Taj et al., 2016). The sections were analyzed at 100× magnification using a TissueFax Plus system coupled with a Zeiss^®^ AxioImagerZ2 Microscope (Jena, Germany). Images were acquired using the TissueFaxs (Tissue-Gnostics^®^, Vienna, Austria) software. The number of GAD65/67^+^ immunostained cells in the largest amygdala area of each section was quantified using HistoQuest^®^ (Tissue-Gnostics) software. The data for each rat were derived from the average of those three sections. The raw data used in the analysis were imported into SPSS 21.0 (IBM, Armonk, NY, USA) for further statistical analysis.

To determine the number of GAD65/67^+^ cells that exhibited p-eIF2α/ATF4/CHOP, cell counting was performed as described previously (Shi et al., 2015). Five rats from each group were used for morphological observation and data analysis. The numbers of p-eIF2α^+^-GAD65/67^+^, ATF4^+^-GAD65/67^+^ and CHOP^+^-GAD65/67^+^ cells were counted at 100× magnification. The average number of positive cells in each rat was calculated by two independent observers who were blinded to the experimental conditions.

### Statistical Methods

The results are presented as the mean ± standard error of the mean (SEM). Data were analyzed using one-way ANOVA followed by a *post hoc* least significant difference (LSD) *t*-test to determine specific group differences. The action of the inhibitor sal was analyzed by two-way ANOVA. Differences between the two experimental groups were compared using student’s *t*-test. All statistical analyses were performed using GraphPad Prism 5 (GraphPad Software Inc., San Diego, CA, USA) and SPSS 21.0. Significance was defined as *P* < 0.05 for all statistical tests.

## Results

### Stress Caused Anxiety and Neurotransmitter Level Changes in the BLA of Rats

**Figure 1:**
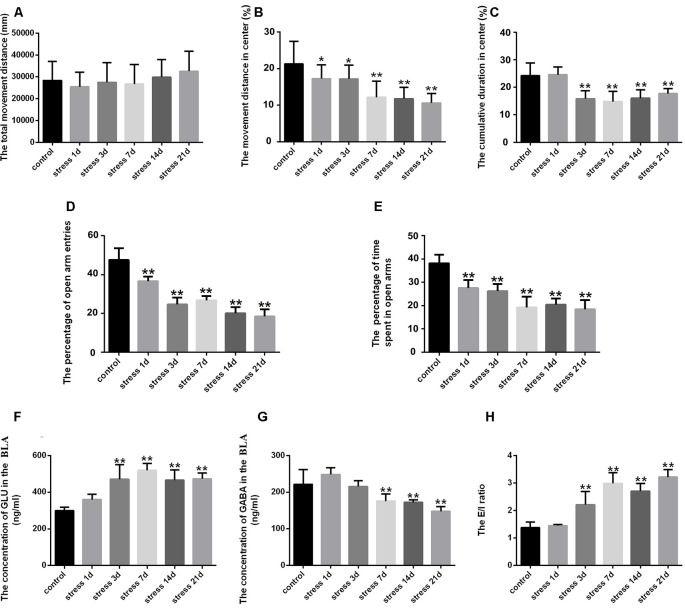
Stress caused anxiety-like behavior and neurotransmitter level changes in the amygdala of rats. **(A)** Total movement distance on the open field test. **(B)** Percentage of movement distance in the center area of the open field test. **(C)** Percentage of cumulative duration in the center area of the open field test. **(D)** Percentage of open arm entries on the elevated plus-maze (EPM). **(E)** Percentage of time spent in open arms of the EPM. **(F)** The concentration of GLU in the amygdala. **(G)** The concentration of γ-aminobutyric acid (GABA) in the amygdala. **(H)** The excitation/inhibition (E/I) ratio. Data (mean ± SEM) represent. *P < 0.05, **P < 0.01 vs. control group (*n* = 10).

The effect of anxiety-like behavior on experimental rats was measured in open field and EPM tests (Yin et al., 2019). For the open field test, ANOVA revealed that stress treatment led to significant differences in the percentage of movement distance in the center area (*F*_(5,54)_ = 10.202; *P* < 0.001) and cumulative duration in the center area (*F*_(5,54)_ = 15.207; *P* < 0.001), while no difference was found in the total movement distance (*F*_(5,54)_ = 0.893; *P* = 0.492). As shown in Figures 1A–C, *post hoc* test indicated that stress treatment resulted in a decrease in the percentage of movement distance (*P* < 0.05) and cumulative duration in the center area (*P* < 0.01). ANOVA of the EPM results also revealed significant changes in the percentage of open arm entries (*F*_(5,54)_ = 99.968; *P* < 0.001) and the percentage of time spent in open arms (*F*_(5,54)_ = 47.546; *P* < 0.001) after stress treatment. The *post hoc* test indicated that the percentage of open arm entries and the percentage of time spent in open arms were significantly lower in the stress groups (*P* < 0.01, *P* < 0.01; Figures 1D,E). All of these results suggested stress could result in anxiety in rats.

GLU and GABA represent excitatory and inhibitory neurotransmitters, respectively, in the BLA, and were detected by HPLC. ANOVA of GLU levels showed significant changes after stress exposure (*F*_(5,24)_ = 15.988; *P* < 0.001). As shown in Figure 1F, compared with the control group (299.81 ± 8.18), the concentration of GLU was markedly increased in the stress group at 3 days (470.53 ± 35.30, *P* < 0.01), 7 days (519.01 ± 17.19, *P* < 0.01), 14 days (465.84 ± 25.00, *P* < 0.01) and 21 days (472.70 ± 14.00, *P* < 0.01), but no difference was detected at 1 day (359.13 ± 13.05, *P* > 0.05). ANOVA of GABA levels showed significant effects of stress exposure (*F*_(5,24)_ = 15.047; *P* < 0.001). As shown in Figure 1G, the GABA concentration was significantly decreased after 7 days (175.45 ± 8.73, *P* < 0.01), 14 days (172.01 ± 3.21, *P* < 0.01) and 21 days (147.76 ± 5.60, *P* < 0.01) of stress exposure compared with the control group (221.33 ± 17.99), although no difference was observed after 1 day (248.09 ± 8.26, *P* > 0.05) or 3 days (215.43 ± 7.26, *P* > 0.05). Stress-induced perturbation of GLU and GABA in the BLA. Moreover, the E/I ratio was significantly increased after 3 days of stress exposure (*P* < 0.01; Figure 1H).

### Stress Damaged the Neurons and Decreased the Number of GABAergic Neurons in the BLA

**Figure 2:**
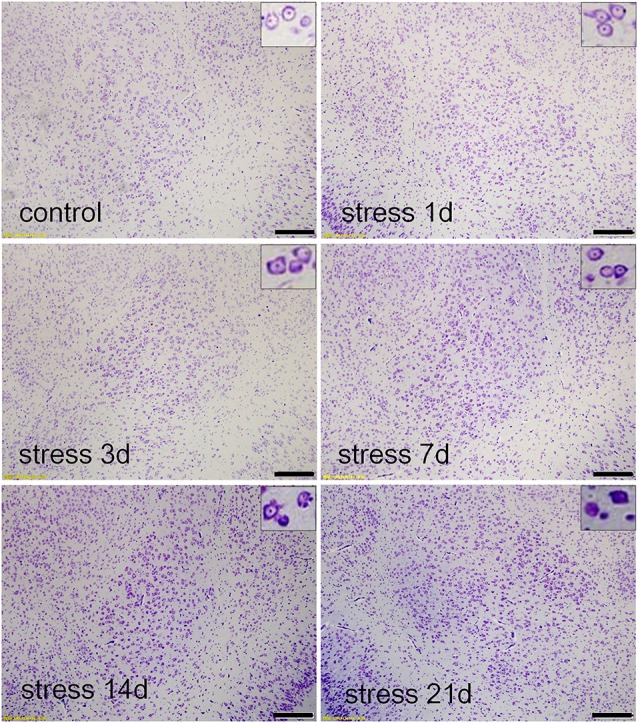
Stress damaged the basolateral amygdala (BLA) neurons. Thionin staining in the control group, and stress groups at 1 day, 3 days, 7 days, 14 daysand 21 days, respectively. High-power photomicrographs in the upper right corners are representative images showing neurons in panels. With increasing periods of stress exposure, Nissl bodies were not clear, neurophagy and pyknotic neurons were visible. Bars = 200 μm.

Thionin staining is a traditional method used to observe pathological changes in neurons (Yi et al., 2019). As shown in Figure 2, the neuronal structures were clear, and Nissl bodies were evenly distributed in the cytoplasm in the control group and the stress group at 1 and 3 days. However, after 7 days of stress exposure edema was observed in the neurons and some Nissl bodies were not clear. With prolonged duration of stress exposure, neurophagy and pyknotic neurons were visible (14 and 21 days).

**Figure 3:**
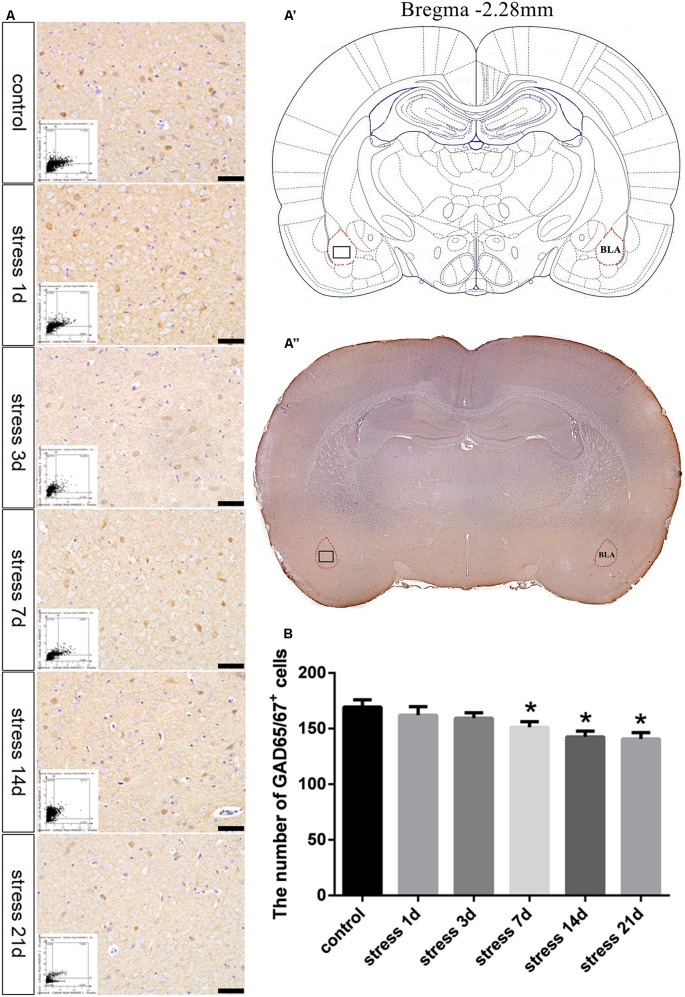
Stress contributed to decrease in the number of GABAergic neurons. **(A)** Representative images showing GAD65/67 immunohistochemistry in the BLA. Images obtained by microscopy-based multicolor tissue cytometry (MMTC) are shown in the lower left corners. Bars = 50 μm. The analyzed region within the BLA is schematically illustrated in **(A′)** (diagram modified from Paxinos and Watson, 2007) and **(A”)** (GAD65/67 immunohistochemistry). **(B)** Quantitative MMTC analysis. The data are shown as mean ± SEM, *P < 0.05 vs. control group (*n* = 5).

GAD65/67 is a member of the group II decarboxylase family of proteins and is responsible for catalyzing the rate-limiting step in the production of GABA. In the present study, the number of GAD65/67^+^ cells represents the number of GABAergic neurons (Figure 3). ANOVA of the numbers of GAD65/67^+^ cells showed a significant difference among the groups (*F*_(5,24)_ = 3.712; *P* = 0.012). No change in the number of GAD65/67^+^ cells was observed in the stress group at 1 day (162.00 ± 7.67, *P* > 0.05) or 3 days (159.40 ± 4.87, *P* > 0.05) compared with the control group (169.40 ± 6.45), but the number of GAD65/67^+^ cells was significantly decreased in the stress group at 7 days (151.20 ± 5.08, *P* < 0.05), 14 days (142.60 ± 5.16, *P* < 0.05) and 21 days (140.80 ± 5.54, *P* < 0.05).

### Inhibition of the PERK Pathway Counteracts Anxiety and Neurotransmitter Changes in the BLA of Rats

**Figure 4:**
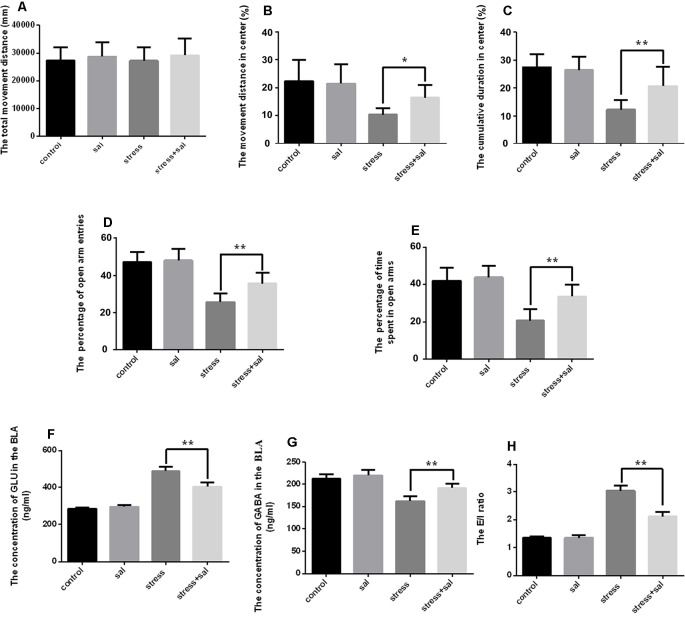
Inhibition of the PERK pathway counteracts anxiety and neurotransmitter changes in the amygdala of rats. **(A)** Total movement distance on the open field test. **(B)** Percentage of movement distance in the center area of the open field test. **(C)** Percentage of cumulative duration in the center area of the open field test. **(D)** Percentage of open arm entries on the EPM. **(E)** Percentage of time spent in open arms of the EPM. **(F)** The concentration of GLU in the amygdala. **(G)** The concentration of GABA in the amygdala. **(H)** The excitation/inhibition (E/I) ratio. Data (mean ± SEM) represent. *P < 0.05, **P < 0.01 vs. stress group (*n* = 10).

To investigate the effect of ERS on anxiety in rats, we applied the PERK pathway inhibitor sal. For the open field test, two-way ANOVA revealed that the interaction between sal and stress treatment and stress treatment alone had significant effects on the cumulative duration in the center area (*F*_(1,36)_ = 8.756, *P* = 0.005; *F*_(1,36)_ = 42.942, *P* < 0.001) and the percentage of movement distance in the center area (*F*_(1,36)_ = 4.473, *P* = 0.041; *F*_(1,36)_ = 19.615, *P* < 0.001), while no significant effect on the total movement distance was observed (*F*_(1,36)_ = 0.034, *P* = 0.854; *F*_(1,36)_ = 0.014, *P* = 0.908). No significant effects of sal treatment on the total movement distance (*F*_(1,36)_ = 1.000, *P* = 0.324) or the percentage of movement distance in the center area were observed (*F*_(1,36)_ = 2.754, *P* =0.106), but an effect on the cumulative duration in the center area was found (*F*_(1,36)_ = 5.308, *P* =0.027). As shown in Figures 4A–C, the *post hoc* test indicated that sal and stress treatment resulted in an increase in the percentage of movement distance (*P* < 0.05) and cumulative duration in the center area (*P* < 0.01). Meanwhile, two-way ANOVA of the EPM results also revealed that the interaction among sal combined with stress treatment, stress treatment, and sal treatment had significant effects on the percentage of open arm entries (*F*_(1,36)_ = 6.777, *P* = 0.013; *F*_(1,36)_ = 94.052, *P* < 0.001; *F*_(1,36)_ = 9.723, *P* = 0.004) and the percentage of time spent in open arms (*F*_(1,36)_ = 7.624, *P* = 0.009; *F*_(1,36)_ = 60.001, *P* < 0.001; *F*_(1,36)_ = 12.903, *P* = 0.001). *Post hoc* test indicated that the percentage of open arm entries and the percentage of time spent in open arms were increased after sal combined with stress treatment (*P* < 0.01, *P* < 0.01; Figures 4D,E). All of these results indicated inhibition of the PERK pathway could relieve anxiety.

We also detected the concentrations of GLU and GABA using HPLC after sal administration. Two-way ANOVA of GLU and GABA levels and the E/I ratio showed significant effects on the interaction among stress combined with sal treatment, stress treatment, and sal treatment (GLU: *F*_(1,36)_ = 43.607, *P* < 0.001; *F*_(1,36)_ = 428.331, *P* < 0.001; *F*_(1,36)_ = 25.893, *P* < 0.001; GABA: *F*_(1,36)_ = 4.823, *P* = 0.043; *F*_(1,36)_ = 64.508, *P* < 0.001; *F*_(1,36)_ = 14.351, *P* =0.002; E/I ratio: *F*_(1,36)_ = 55.427, *P* < 0.001; *F*_(1,36)_ = 380.539, *P* < 0.001; *F*_(1,36)_ = 53.556, *P* < 0.001). *Post hoc* test revealed that the levels of GLU, GABA and E/I ration showed significant changes after sal pretreatment stress expose (*P* < 0.01, *P* < 0.01, *P* < 0.01; Figures 4F,G,H). All these results suggested that inhibition of the PERK pathways could counteract the changes of neurotransmitter in the BLA of rats.

### Inhibition of the PERK Pathway Alleviated GABAergic Neuron Injury in the BLA

**Figure 5:**
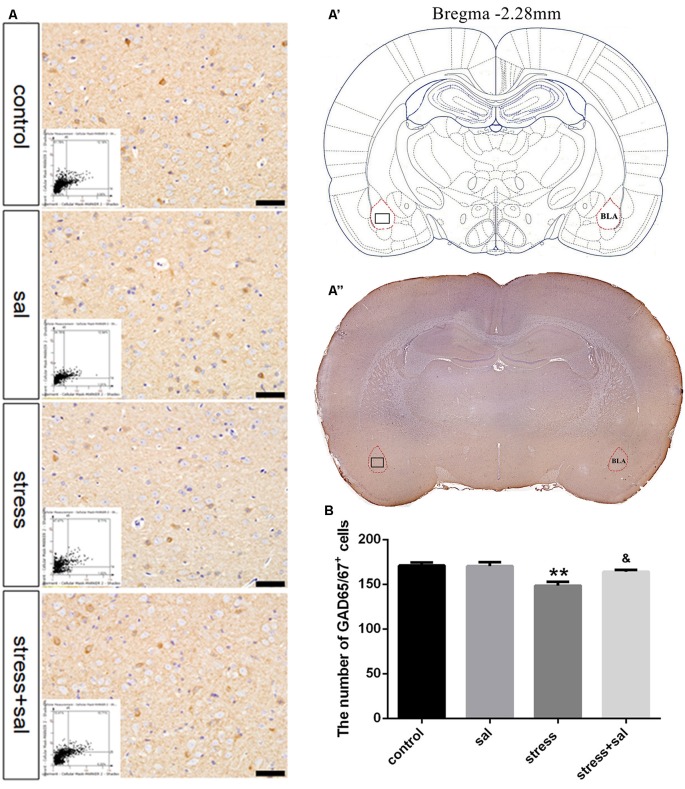
Inhibition of the PERK pathway alleviated GABAergic neuron injury. **(A)** Representative images showing GAD65/67 immunohistochemistry in the BLA. Images obtained by MMTC are shown in the lower left corners. Bars = 50 μm. The analyzed region within the BLA is schematically illustrated in **(A′)** (diagram modified from Paxinos and Watson, 2007) and **(A”)** (GAD65/67 immunohistochemistry). **(B)** Quantitative MMTC analysis. Data are shown as mean ± SEM, **P < 0.01 vs. control group, ^&^*P* < 0.05 vs. stress group (*n* = 5).

Representative images of immunohistochemical staining are shown in Figures 5A–A”. HistoQuest^®^ software was used to quantify the number of GAD65/67^+^ cells in the BLA (Figure 5B). Two-way ANOVA revealed that stress treatment (*F*_(1,16)_ = 14.939, *P* = 0.001) and the interaction between stress and sal treatment (*F*_(1,16)_ = 4.611, *P* = 0.047) had significant effects on GAD65/67^+^ cells, whereas no significant effect was observed for sal treatment (*F*_(1,16)_ = 3.945, *P* = 0.064). The *post hoc* test showed there was a decrease (*P* < 0.01) in the number of GAD65/67^+^ cells in the BLA after stress treatment. In contrast, sal and stress treatment resulted in an increase (*P* < 0.05) of the number of GAD65/67^+^ cells in the BLA.

### p-eIF2α, ATF4, and CHOP Protein Expression in the BLA

**Figure 6:**
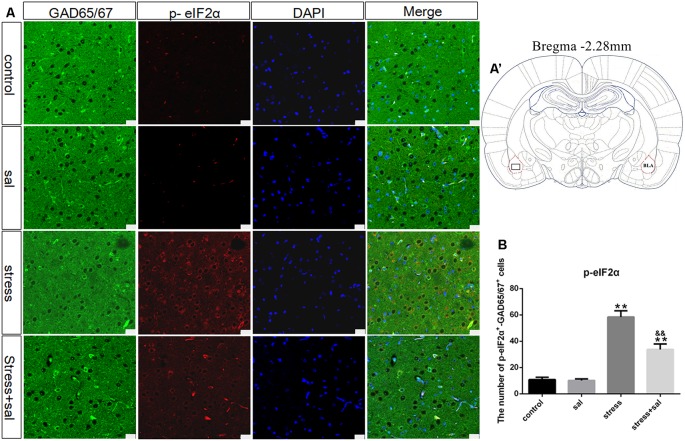
Number of GAD65/67 positive neurons expressing p-eIF2α in the BLA. **(A)** Representative confocal images showing coronal sections of rat immunostained for GAD65/67 (green), p-eIF2α (red), DAPI (nuclear stain, blue) and merged (orange/yellow). Bars = 25 μm. The analyzed region within the BLA is schematically illustrated in **(A′)** (diagram modified from Paxinos and Watson, 2007). **(B)** Quantitative analysis of the number of p-eIF2α^+^-GAD65/67^+^ cells (one-way ANOVA). Data are shown as mean ± SEM, **P < 0.01 vs. control group, ^&&^*P* < 0.01 vs. stress group (*n* = 5).

**Figure 7:**
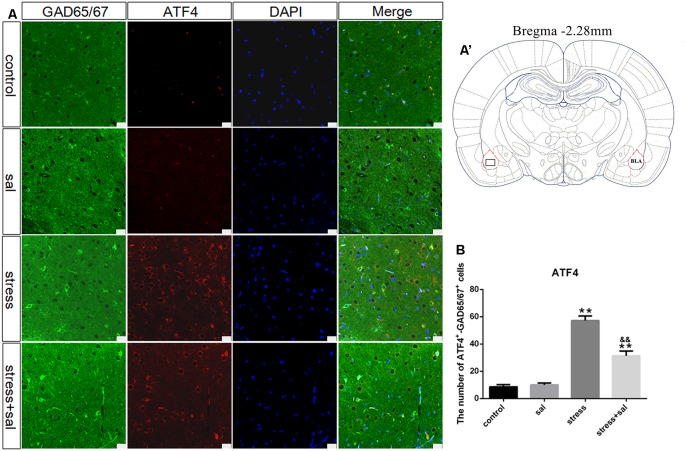
Number of GAD65/67 positive neurons expressing ATF4 in the BLA. **(A)** Representative confocal images showing coronal sections of rat immunostained for GAD65/67 (green), ATF4 (red), DAPI (nuclear stain, blue) and merged (orange/yellow). Bars = 25 μm. The analyzed region within the BLA is schematically illustrated in **(A′)** (diagram modified from Paxinos and Watson, 2007). **(B)** Quantitative analysis of the number of ATF4^+^-GAD65/67^+^ cells (one-way ANOVA). Data are shown as mean ± SEM, **P < 0.01 vs. control group, ^&&^*P* < 0.01 vs. stress group (*n* = 5).

**Figure 8:**
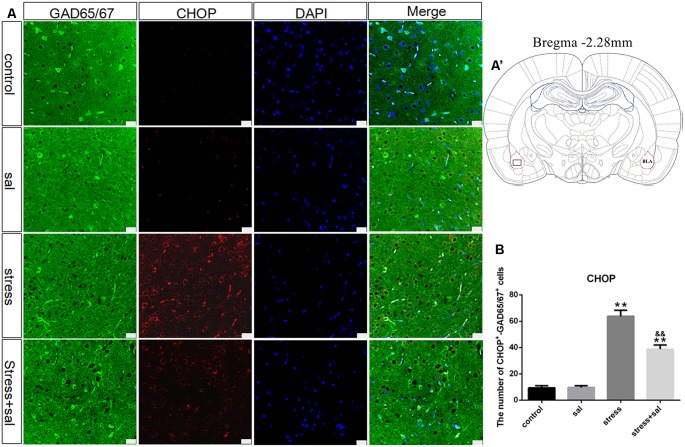
Number of GAD65/67 positive neurons expressing CHOP in the BLA. **(A)** Representative confocal images showing coronal sections of rat immunostained for GAD65/67 (green), CHOP (red), DAPI (nuclear stain, blue) and merged (orange/yellow). Bars = 25 μm. The analyzed region within the BLA is schematically illustrated in **(A′)** (diagram modified from Paxinos and Watson, 2007). **(B)** Quantitative analysis of the number of CHOP^+^-GAD65/67^+^ cells (one-way ANOVA). Data are shown as mean ± SEM, **P < 0.01 vs. control group, ^&&^*P* < 0.01 vs. stress group (*n* = 5).

Double-labeling experiments showed that the p-eIF2α, ATF4, and CHOP proteins were located in the cytoplasm and colocalized with the GABAergic neuron marker GAD65/67^+^ in the BLA (Figures 6A–A’, 7A–A’, 8A–A’). Two-way ANOVA of p-eIF2α^+^-GAD65/67^+^ cells revealed main effects for stress treatment (*F*_(1,16)_ = 112.454, *P* < 0.001), sal treatment (*F*_(1,16)_ = 14.087, *P* = 0.002) and a significant interaction between sal and stress treatment (*F*_(1,16)_ = 12.777, *P* = 0.003). As shown in Figure 6B, *post hoc* test indicated that there was an increase in the number of p-eIF2α^+^-GAD65/67^+^ cells after stress treatment (*P* < 0.01) or sal and stress treatment (*P* < 0.01). In contrast, sal and stress treatment decreased (*P* < 0.01) the number of p-eIF2α^+^-GAD65/67^+^ cells in the BLA. Two-way ANOVA showed a significant modification in ATF4^+^-GAD65/67^+^ cells occurred after stress treatment (*F*_(1,16)_ = 170.970, *P* < 0.001), sal treatment (*F*_(1,16)_ = 20.773, *P* < 0.001) and sal add stress treatment (*F*_(1,16)_ = 25.814, *P* < 0.001). As shown in Figure 7B, *post hoc* comparisons showed a significant increase in the number of ATF4^+^-GAD65/67^+^ cells after stress treatment (*P* < 0.01) or sal and stress treatment (*P* < 0.01). In contrast, sal add stress treatment decreased (*P* < 0.01) the number of ATF4^+^-GAD65/67^+^ cells in the BLA. Two-way ANOVA of CHOP^+^-GAD65/67^+^ cells showed significant effects of stress treatment (*F*_(1,16)_ = 201.411, *P* < 0.001), sal treatment (*F*_(1,16)_ = 17.724, *P* < 0.001) and sal and stress treatment (*F*_(1,16)_ = 18.301, *P* < 0.001). *Post hoc* test indicated that there was an increase in the number of CHOP^+^-GAD65/67^+^ cells during stress treatment (*P* < 0.01) or sal and stress treatment (*P* < 0.01). In contrast, sal add stress treatment resulted in a decrease (*P* < 0.01) in the number of CHOP^+^-GAD65/67^+^ cells (Figure 8B).

## Discussion

Stress is essentially a defensive adaptive response used by the body to maintain homeostasis of the internal environment through neuroendocrine regulation to better adapt to changes in external stimuli. However, when stimuli exceed the ability of homeostasis regulation, the body will exhibit various functional and metabolic disorders, leading to different degrees of damage (Marin et al., 2011). Previous studies have indicated that prolonged stress could result in various mental disorders, including anxiety, depression, and other neuropsychiatric diseases (Goldstein, 2011). A rat model of restraint and forced ice-water swimming is an ideal model that well reflects the effect of complex physical and psychological stress on the body (Yi et al., 2017, 2019). In this study, we established rat models with different durations of restraint and forced ice-water swimming and found that the rats showed obvious disorders, such as anxiety, with prolongation of stress exposure.

The amygdala is an important part of the limbic system and plays a critical role in stress-induced mental disorders (LeDoux, 2000; Davis and Whalen, 2001). The BLA, an important subnucleus of the amygdala, initiates and determines the response of the amygdala to stress events *via* glutamatergic projections to the CeA. A disturbance in the balance of excitation and inhibition is the main cause of mental disorders, including anxiety (Kent et al., 2002), and is mainly manifested by obvious upregulation of the excitatory neurotransmitter GLU and significant downregulation of the inhibitory neurotransmitter GABA (Davis et al., 1994; Pitsikas, 2014). Similarly, a disturbance of the excitatory and inhibitory neurotransmitter balance in the BLA also causes anxiety in rats, which may be due to changes in the glutamatergic projection intensity from the BLA to the CeA induced by stress exposure. Although GABAergic neurons comprise a minority of neurons in the BLA (20%; Davis et al., 1994; Chauveau et al., 2012), changes in these neurons affect glutamatergic projections. In the present study, we observed BLA neurons with thionin staining. The results revealed that with prolonged stress exposure, Nissl bodies disappeared, pyknotic neurons were observed, and BLA neuron injury was obvious. Moreover, we also detected the number of GABAergic neurons labeled by a specific marker, GAD65/67, and the results showed that GABAergic neurons were significantly decreased after long-term stress exposure. The decrease in GABA in the BLA was due to the decreasing number of GABAergic neurons. Most GABAergic neurons are locally projecting neurons that regulate the neurotransmission of adjacent cells (de la Cadena et al., 2014); therefore, the decreased inhibitory effect of GABA on glutamatergic neurons induced by stress could result in a significant increase in GLU in the case of BLA neuron injury. In addition, in spite of some impaired glutamatergic neurons in the BLA during stress, the overexcitation of the remaining glutamatergic neurons may be another reason for the increase of GLU.

The ER is an important organelle for protein synthesis, glycosylation, folding, and secretion, as well as nascent protein transport, which is the basis for cell survival and maintenance of normal function (Nakayama et al., 2008). Alterations in ER function by intracellular or extracellular stimuli cause ERS, which leads to the accumulation of unfolded and misfolded proteins in the ER lumen and activates an adaptive response commonly referred to as the UPR. PERK, as a transmembrane protein of the ER, has been shown to be a vital receptor in the UPR (Hetz, 2012). Normally, PERK binds to glucose-regulated protein 78 (GRP78) to form a complex. When the UPR is initiated, the accumulating unfolded and misfolded proteins cause dissociation of GRP78 from PERK, allowing PERK activation. Activated PERK directly phosphorylates eIF2α (Ron, 2002), inhibits mRNA transcription (Lu et al., 2004), and reduces the load of newly synthesized proteins (Hetz, 2012). Meanwhile, phosphorylated eIF2α also promotes the translocation and activation of ATF4. The UPR is a relatively conservative and protective ERS response in biological evolution, but in the case of strong stimulation, the ERS-induced UPR can lead to cell death *via* the activation of the PERK-eIF2α-ATF4-CHOP pathway. This pathway selectively induces the transcription of ATF4 and promotes its binding to the amino acid response element (AARE), thereby inducing the expression of CHOP (Oyadomari and Mori, 2004). Increasing evidence indicates that ERS not only participates in the pathogenesis of dysfunction caused by ischemia and hypoxia but is also involved in the process of cell death induced by various neurodegenerative diseases (Puri and Morris, 2018; Deng et al., 2019). The results of this study indicated that stress could damage GABAergic neurons and decrease the number of neurons in the BLA, but the specific mechanism of this damage is still unclear. To further determine whether ERS is involved in the injury process, we detected ERS-related proteins in rats exposed to stress for 7 days (all changes were obvious after stress exposure for 7 days, especially GABAergic neuron injury). We found that p-eIF2α, ATF4, and CHOP were significantly increased in GABAergic neurons in the BLA, and the ERS PERK-eIF2α-ATF4-CHOP pathway was activated. Additionally, for further verification, we used sal to inhibit the ERS PERK pathway and investigated the changes in the expression of neurotransmitters, the number of GABAergic neurons, and ERS-related proteins in the BLA after inhibition. Sal is an ERS inhibitor that acts by selectively inhibiting eIF2α dephosphorylation and plays a protective role in cells (Boyce et al., 2005; Yuan et al., 2019). Our results also confirmed that expression of the ERS proteins p-eIF2α, ATF4, and CHOP was significantly decreased after application of sal, and the number of GABAergic neurons was obviously increased. Sal alleviated the damage to GABAergic neurons of the BLA induced by stress and increased GABA release, which decreased the GLU content by enhancing the inhibition of glutamatergic neurons in the BLA. Thereby, these changes can eventually reduce the intensity of glutamatergic projections from the BLA to the CeA and ameliorate mental disorders such as anxiety in rats.

In conclusion, stress resulted in neurotransmitter alterations in the BLA and obvious anxiety disorders in rats. Moreover, ERS-mediated GABAergic neuron injury in the BLA, is an important cause of neurotransmitter alterations, which are closely related to stress-induced mental disorders.

## Data Availability Statement

All datasets generated for this study are included in the article.

## Ethics Statement

The animal study was reviewed and approved by the Institutional Review Board for Animal Experiments at Hebei Medical University.

## Author Contributions

SW, WS, GZ, XZ, CM, KZ, BC and YL wrote the article, designed and performed the experiments. SW, WS and GZ did the stats and organized the data. XZ and KZ created the figures. CM, BC and YL supervised the research design and revised the manuscript. All authors read and commented on the manuscript.

## Conflict of Interest

The authors declare that the research was conducted in the absence of any commercial or financial relationships that could be construed as a potential conflict of interest.
